# Accelerated nucleation of the 2014 Iquique, Chile Mw 8.2 Earthquake

**DOI:** 10.1038/srep24792

**Published:** 2016-04-25

**Authors:** Aitaro Kato, Jun’ichi Fukuda, Takao Kumazawa, Shigeki Nakagawa

**Affiliations:** 1 Earthquake and Volcano Research Center, Graduate School of Environmental Studies, Nagoya University, Nagoya, Japan, Furo-cho, Chikusa-ku, Nagoya, 464-8601, Japan; 2 Earthquake Research Institute, University of Tokyo, Tokyo, Japan, 1-1-1 Yayoi, Bunkyo-ku, Tokyo, 113-0032, Japan; 3 The Institute of Statistical Mathematics, Tachikawa, Japan, 10-3 Midori-cho, Tachikawa, Tokyo, 190-8562, Japan

## Abstract

The earthquake nucleation process has been vigorously investigated based on geophysical observations, laboratory experiments, and theoretical studies; however, a general consensus has yet to be achieved. Here, we studied nucleation process for the 2014 Iquique, Chile Mw 8.2 megathrust earthquake located within the current North Chile seismic gap, by analyzing a long-term earthquake catalog constructed from a cross-correlation detector using continuous seismic data. Accelerations in seismicity, the amount of aseismic slip inferred from repeating earthquakes, and the background seismicity, accompanied by an increasing frequency of earthquake migrations, started around 270 days before the mainshock at locations up-dip of the largest coseismic slip patch. These signals indicate that repetitive sequences of fast and slow slip took place on the plate interface at a transition zone between fully locked and creeping portions. We interpret that these different sliding modes interacted with each other and promoted accelerated unlocking of the plate interface during the nucleation phase.

Subduction of the Nazca plate beneath the South American plate, at an average rate of ~7 cm/yr, has produced a series of megathrust subduction earthquakes along the Chile–Peru Trench (Fig. 1). The mainshock rupture of the 2014 Iquique, Chile Mw 8.2 earthquake occurred within the North Chile seismic gap, which stretches for ~500 km along the plate boundary^1^. The last great earthquake in this region (an estimated Mw 8.8 event) took place 137 years ago. Much of the fault is currently fully locked^2^,^3^; however, the 2014 mainshock only partly released the accumulated strain energy of the seismic gap^1^,^4^,^5^,^6^.

A recent deployment of modern seismic and geodetic instrumentation along the northern Chile coast has provided an excellent opportunity to explore the nucleation process of megathrust earthquakes. According to previous studies, the mainshock was preceded by intensive foreshock sequences and geodetic signals lasting at least two weeks, showing gradual unlocking of the plate boundary fault updip of the largest coseismic slip patch^5^,^7^,^8^,^9^,^10^,^11^. However, the long-term behaviors of the plate boundary fault before the mainshock rupture remain poorly understood.

Because seismicity is one of the most powerful tools for identifying nucleation processes, it is essential to investigate the spatial–temporal evolution of earthquakes leading up to the 2014 mainshock rupture^8^,^11^. In this work, to more precisely characterize the long-term earthquake behavior, we searched the continuous waveforms for events with similar seismograms to those of each template event, applying a matched filter technique to data recorded near the source region from 1 January 2008 to 31 May 2014 (see Supplementary Fig. S1 online). This technique provided us with a new decadal-scale earthquake catalog with more uniform and complete detections than pre-existing catalogs^12^,^13^,^14^,^15^ (Supplementary Figs S2 and S3). Our new catalog included several repeating earthquakes (Supplementary Fig. S4), which are recognized as a “creep meter” because they indicate the evolution of aseismic slip transients along the plate boundary^16^,^17^,^18^,^19^,^20^,^21^,^22^. To identify repeating earthquakes as accurately and completely as possible, we focused on three key observations: waveform similarity in a long time window, magnitude difference, and approximate colocation of each event pair. These constraints are necessary to ensure that two repeating earthquakes rupture nearly identical patches.

Finally, we computed the time-dependent rate of background seismicity by applying the non-stationary epidemic-type aftershock sequence (ETAS) model^23^,^24^ to the earthquake catalog. Previous studies found that changes in the background rate of seismicity have a good correlation with transient aseismic processes such as slow slip events and fluid intrusions^23^,^25^,^26^. Our combined analysis of the spatial–temporal evolution of detected seismicity, repeating earthquakes, and the background rate of seismicity provides us with new insights into the nucleation stage of the 2014 Chile earthquake, which possibly initiated around 270 days before the mainshock failure.

## Results

We identified 11690 earthquakes, which is nearly 17 times the number of available template events listed in the USGS catalog (684 events) (Supplementary Fig. S2), showing a significant improvement in catalog completeness. The magnitude of completeness of the detected events is around 3.8 before the mainshock (Supplementary Fig. S3). The space–time diagram of the newly detected events shows that the seismicity rate off Iquique was almost constant before the summer of 2013 with a high ratio of the background seismicity (Fig. 2a,b). Similarly, the rate of aseismic slip derived from repeating earthquakes was nearly constant at ~0.6 cm/yr (Fig. 2c), which is an order of magnitude lower than the convergent rate of the Nazca plate. This low rate of increase in aseismic slip is consistent with geodetic measurements, which suggest that the northern Chile seismic gap is fully locked as a whole^2^,^3^.

In the summer of 2013, an intensive seismic swarm occurred around the southern edge and updip of the largest slip patch of the mainshock rupture (Figs 2a and 3a). Some repeating earthquakes simultaneously occurred during the seismic swarm, indicating concurrent episodic aseismic slip (Figs 2c and 3b). Similarly, the cumulative number of background seismicity showed a slight upward deviation from the linear trend (Figs 2b and 3c). This seismic swarm showed bilateral migration of earthquakes with a speed of ~1 km/day (inset in Fig. 2a). The increase in aseismic slip during the swarm, averaged over the entire region (using all groups of the repeating earthquakes), was ~0.7 cm. Following the seismic swarm, the seismicity rate, the amount of aseismic slip, the background rate of seismicity, and the frequency of earthquake migrations episodically accelerated during the period approaching the mainshock failure (Figs 2 and 3). Thus, the seismic swarm during the summer of 2013 was probably the first indicator of initial unlocking of the plate boundary fault^27^.

A second crisis of seismic bursts occurred at the southern and northern edges of the mainshock rupture area between January and February 2014^8^. During the early part of the February 2014 seismic burst, we identified southward migrations of earthquakes at a rate of ~10 km/day at the northern edge, accompanied by repeating earthquakes (inset in Fig. 2a) and a further increase in the background rate of seismicity (Fig. 3c). The total increase in the measured average displacement of aseismic slip was ~1.3 cm during this second crisis.

Following the reactivation of the northern edge of the rupture zone on 15 March 2014, the final seismic burst was characterized by intense multiple earthquake migrations during the periods 15–17 March, 16–18 March, and 22–24 March 2014, which extensively developed within the entire foreshock zone^8^,^11^ (Supplementary Fig. S5). These earthquakes migrated bilaterally in the along-strike and down-dip directions at speeds of 2–10 km/day (Fig. 3d). The seismicity included many repeating earthquakes, resulting in a sharp increase in aseismic slip (up to ~8.0 cm) during the final seismic crisis (Figs 3b and S5). In addition, the onset of the final two seismic swarms (16–18 March and 22–24 March 2014) was correlated with the final increase in the background rates of seismicity (Fig. 3c)^5^, although the increase in the background seismicity was smoother, compared with that of the seismicity or the amount of aseismic slip, due to a temporal smoothness constraint employed in the inversion of the background rate of seismicity.

## Discussions

From our expanded earthquake catalog (Figs 2 and 3), we inferred that the first crisis leading to the mainshock rupture started in summer 2013, accompanied by migrating seismic bursts at the southern edge of the largest coseismic slip patch. Following this burst, multiple repetitive sequences of migrating seismic bursts took place along dip as well as along strike, outlining the shallow rim of the largest coseismic slip patch. The frequency of earthquake migrations increased, and the migration speeds also tended to rise before the mainshock failure (Fig. 3d). This migrating behavior is consistent with a numerical modeling work of Ariyoshi *et al.*^28^, which showed that very low-frequency earthquakes had higher migration speeds and shorter recurrence intervals during the nucleation stage of a simulated megathrust earthquake.

These migrating seismic bursts were accompanied by the transient increase in aseismic slip and the background rate of seismicity (Figs 2 and 3). Furthermore, the lateral size of each seismic swarm area was considerably larger than the earthquake source dimensions. Based on these observations, a likely explanation for the earthquake migrations is the propagation of episodic aseismic slip transients; i.e., slow slip events, at the transition zone from full coupling to creep^8^,^11^,^14^,^29^,^30^. However, we cannot rule out the alternative interpretation that these earthquake migrations resulted from a succession of independent afterslip sequences following each earthquake^31^.

During the final 17 days before the mainshock (15–31 March, 2014), the continuous GPS stations located along the coast near the source region started to move trench-ward. These surface deformations resulted from the gradual unlocking of the plate interface^5^,^9^. However, it is debated whether the unlocking of the plate interface was driven mainly by aseismic slip^9^ or cumulative seismic slip from M 5–6 class foreshocks along the plate interface^5^,^10^. This uncertainty arises because the detected surface deformations were too weak to be clearly separated into coseismic and aseismic slip. The observed surface deformations during the final 17 days before the mainshock (15–31 March, 2014) reached up to ~15 mm, even at the GPS station located closest to the epicenter (black arrows in Fig. 4)^10^. Bedford *et al.*^10^ predicted cumulative surface deformations generated by foreshocks, taking into account the uncertainties of focal mechanisms and the elastic constants of a half-space model (blue arrows in Fig. 4). The predicted coseismic displacements are systematically smaller than those observed at the four stations located close to the foreshock area. Therefore, these discrepancies are likely to be explained by transient aseismic slip during the foreshock sequence.

We calculated surface deformations produced by the repeater-inferred aseismic slip (red arrows in Fig. 4), assuming that the aseismic slip took place on the plate interface and occurred parallel to the slip direction of the mainshock rupture. The surface deformations calculated from repeater-inferred slip appear to fill the systematic gaps between the observed and predicted coseismic slip, if we average the cumulative slip of different groups of repeaters in 0.15° × 0.15° grid cells. Based on the repeater-inferred aseismic slip distribution, the total aseismic moment was calculated to be 1.23e + 19 Nm or the equivalent of an Mw 6.7 earthquake, using the shear modulus of 35 GPa. Care must be taken in interpreting the results because repeater-inferred surface deformations and aseismic moment are dependent on grid cell size^11^, although we note that our estimates are on the same order for a reasonable range of grid sizes.

The observation that the same order of cumulative slip was released by seismic and aseismic processes indicates that the transition zone from full coupling to creep was the site of both fast- and slow-slip behaviors during the foreshock sequence^11^. Mixed seismic–aseismic slip behaviors have been recently observed by seismic and geodetic data along a weakly coupled part of the Andean subduction zone in northern Peru^32^. We interpret this to mean that the repeating sequences of fast and slow slip may have interacted with each other and promoted accelerated unlocking of the plate interface during the nucleation phase of the 2014 Iquique earthquake. Subsequently, the accelerated unlocking effectively caused down-dip stress loading on the largest slip patch of the mainshock rupture as the stress level approached the rupture threshold, thereby promoting the mainshock rupture.

The unlocking of plate interface episodically accelerated according to a power-law time-to-failure equation, from summer 2013 to the mainshock failure (Supplementary Fig. S6). In laboratory experiments, fault displacements smoothly increased according to a power-law time-to-failure equation during the nucleation stage^33^, which is analogous to the observations of the present study. However, the evolution of slip is more episodic and irregular in nature than in laboratory experiments^34^. This may indicate the plate boundary fault is more heterogeneous than that in laboratory, and bringing up an importance of investigating the heterogeneity of fault zone structure incorporating interactions between seismic patches with different spatial scales^35^.

It is commonly reported that both fast and slow slip took place surrounding the margin of the largest coseismic slip patch prior to the 2011 Mw 9.0 Tohoku-Oki earthquake, as observed on daily, monthly and decadal time scales^14^,^21^,^36^,^37^,^38^. In particular, a decadal acceleration of the unlocking of the plate interface off-shore of the Tohoku region was observed over a wide region along the down-dip part of the largest coseismic slip region^37^. In contrast, the duration of the accelerated unlocking before the 2014 Iquique earthquake was around 270 days, which is one order of magnitude shorter than that for the Tohoku-Oki event. This large difference in the characteristic time to failure might reflect the scale dependency of critical slip displacement, or spatial variations in fault strength and effective normal stress^33^,^39^.

The seismic potential for megathrust earthquakes within the North Chile seismic gap remains high^1^,^4^,^5^. It is of crucial importance to continuously monitor seismicity, repeating earthquakes, geodetic signals and background rate of seismicity in regions of transition from full coupling to creep, using dense seismic and geodetic instrumentations, in order to detect the nucleation phase of the next “Big One”.

## Methods

### Detection of earthquakes using the matched filter technique

**Figure 1:**
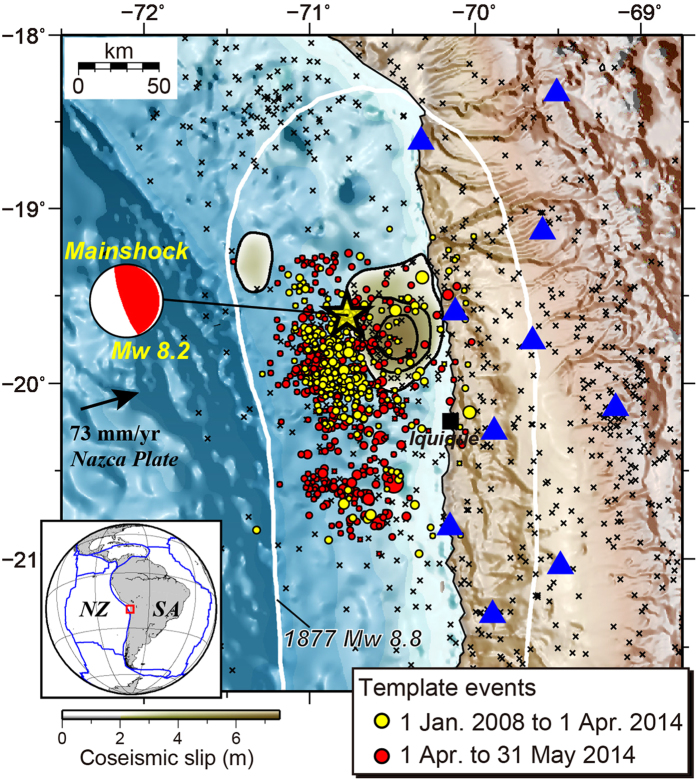
Tectonic setting of the 2014 Iquique, Chile Mw 8.2 earthquake. The yellow star denotes the epicenter of the mainshock with the moment tensor solution by the USGS. The color scale and black contour lines show the coseismic slip distribution estimated by teleseismic waveform inversion^1^. Yellow and red circles are epicenters of matched filter template events before and after the mainshock, respectively. Crosses show USGS catalog epicenters from 1 January 1990 to 31 May 2014. Seismic stations are indicated by blue triangles. The white outline denotes the approximate rupture area of the 1877 Mw 8.8 earthquake. The inset shows the location of the studied region. NZ: Nazca Plate, SA: South American Plate. Bathymetric data are from ETOPO1^50^. Map was created using the GMT (Generic Mapping Tools, http://gmt.soest.hawaii.edu/) software package^51^.

We used continuous three-component velocity seismograms obtained by 10 broadband seismic stations located near the source region (Fig. 1), which are operated and archived by the GFZ – German Research Centre for Geosciences, Institut de Physique du Globe de Paris, Centro Sismológico National, Universidad de Chile, and Universidad Cátolica del Norte, Antofagasta, Chile. Data were continuously recorded by each station at a sampling rate of 100 Hz.

To more precisely characterize the evolution of seismicity prior to the mainshock, we searched for events with similar waveforms to those of each template event, using a matched filter technique^12^,^13^,^14^,^15^. Both continuous data and template waveforms were bandpass filtered from 1 to 8 Hz and decimated to 25 Hz. As template events, we used the earthquakes listed in the USGS earthquake catalog between 1 January 2008 and 31 May 2014. A synthetic S-wave arrival time for each template event was calculated using the one-dimensional seismic velocity structure (Supplementary Fig. S7) derived from a series of seismic surveys conducted near the source region^40^,^41^. To obtain template event waveforms and target waveforms, we used a 10.0 s window, beginning 5.0 s prior to the synthetic S-wave arrival time. We computed correlation coefficients between a template event waveform and a target waveform using incremental detection-window shifts of 0.04 s. At each time step, we calculated the average correlation coefficients over the seismic network whenever the total number of available station channels was greater than 15. We set a threshold for event detection equal to 10 times the MAD (median absolute deviation) of the average correlation coefficients calculated throughout the day of interest. To remove multiple detections, we assigned the location of the detected event to that of the template event with the highest correlation coefficient within the ±5 s window. Then, we computed the magnitude of the detected event based on the median value of the maximum amplitude ratios for all channels between the template and detected events, assuming that a tenfold increase in amplitude corresponds to one unit increase in magnitude^13^. We finally identified 11690 earthquakes, which is nearly 17 times the number listed in the USGS catalog (684 events) (Supplementary Fig. S1). The magnitude of completeness of the detected events is around 3.8 before the mainshock. For more details of the matched filter technique, see Kato *et al.*^15^.

### Extraction of repeating earthquakes

We looked for repeating earthquakes from the newly detected catalog using the waveform data for earthquakes of M 2 or greater. To identify repeating earthquakes as accurately and completely as possible for inferring aseismic slip behavior, we focused on three key observations: waveform similarity in long time windows, magnitude difference, and nearly identical locations. These constrains are necessary to ensure that two repeating earthquakes come from the same asperity.

We selected earthquake pairs with epicenter separations of ≤50 km and calculated cross-correlation coefficients using passband-filtered vertical component seismograms. We used four different frequency bands, as follows: (f_low,_ f_high_) = (0.25–2.0 Hz), (0.5–4.0 Hz), (1.0–8.0 Hz), and (2.0–16.0 Hz). The time window of each seismogram extended from 5.0 s before the P-wave arrival to 15.0 s after the S-wave arrival, which was long enough to include direct and coda phases. We selected candidates of repeating earthquake pairs as those whose cross-correlation coefficients were ≥0.95 at two or more stations in one frequency band, roughly corresponding to the corner-frequency inferred from the average magnitude of each event pair assuming a circular patch model with a constant stress drop of 3 MPa^42^ (Supplementary Table S1), and whose magnitudes (i.e., the average difference in the logarithms of the maximum absolute amplitudes) differed by ≤0.5. The static stress drop during the 2014 Iquique mainshock rupture was estimated to be ~2.5 MPa from finite-fault inversion of teleseismic waves^43^, which is close to stress drops of standard interplate earthquakes. We therefore consider 3 MPa to be a suitable stress drop for analyzing repeating earthquakes in the studied region.

In addition, we are interested in the inter-event distance *d* between earthquake pairs to restrict our analysis to co-located earthquakes. We estimated the inter-event distance *d* for each earthquake pair based on average differential S–P travel time information at sub-sample precision, obtained by waveform cross-correlation^17^,^22^. We determined the differential S–P travel time using an 8.0 s window length beginning 4.0 s before the arrival of each phase. The S-wave seismogram was filtered in the same passband range as the detection windows, while the P-wave seismogram was filtered in the passband range (f_low_*1.732, f_high_*1.732) due to the richness of high-frequency content in the P-wave seismograms. We calculated the largest differential S–P arrival times over the seismic network, all of which had a normalized cross-correlation coefficient of ≥0.75. Assuming typical P- and S-wave velocities for subducting oceanic crust (6.8 and 3.77 km/s, respectively), we computed *d* by multiplying the largest differential S–P time by 8.5 km/s. We compared these *d* with the rupture radii R1 and R2 of the two earthquakes estimated from a circular patch model^44^ with a constant stress drop of 3 MPa. For two earthquakes to be repeating instances of a nearly identical rupture, we required *d *≤ max {R1, R2}. Finally, we grouped together all pairs of repeating earthquakes that share a common earthquake, resulting in 163 groups of 408 repeating earthquakes in the magnitude range 2.0 to 4.6. Supplementary Figure S4 shows an example of waveforms for a group of repeating earthquakes that was active before and after the 2014 Iquique, Chile earthquake.

### Estimation of aseismic slip amount

**Figure 2:**
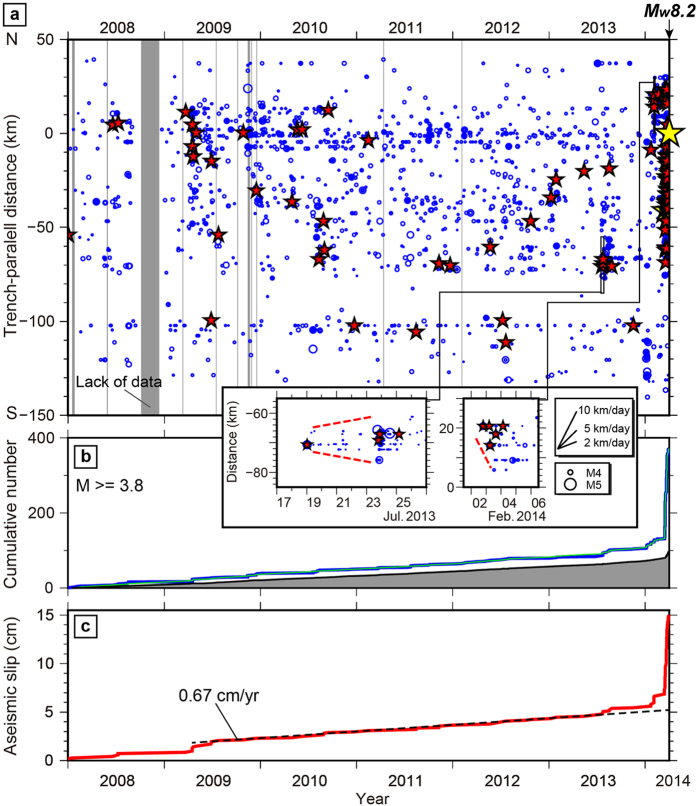
Decadal evolution of seismicity revealed by matched filter processing. (**a**) Space–time diagram of all detected events (blue circles) before the 2014 Iquique, Chile Mw 8.2 earthquake, from 1 January 2008. Red stars indicate repeating earthquakes. Yellow star denotes the hypocenter of the mainshock. The diagram shows earthquake origin times and locations projected onto the N–S strike direction of the mainshock. Periods of insufficient data are indicated by gray vertical bars (Supplementary Fig. S1). The insets show an expanded view of two seismic bursts in July 2013 and February 2014. Red dashed lines represent the approximate locations of the fronts of earthquake migrations. (**b**) Observed (blue thick line) and ETAS-modeled (green line) cumulative number of earthquakes with magnitude ≥3.8. Black line denotes temporal variation of cumulative number of background seismicity (grey shaded area). (**c**) Cumulative displacement of aseismic slip deduced from all groups of the repeating earthquakes. The black dashed line is the best-fit model for the period from March 2009 to July 2013.

**Figure 3:**
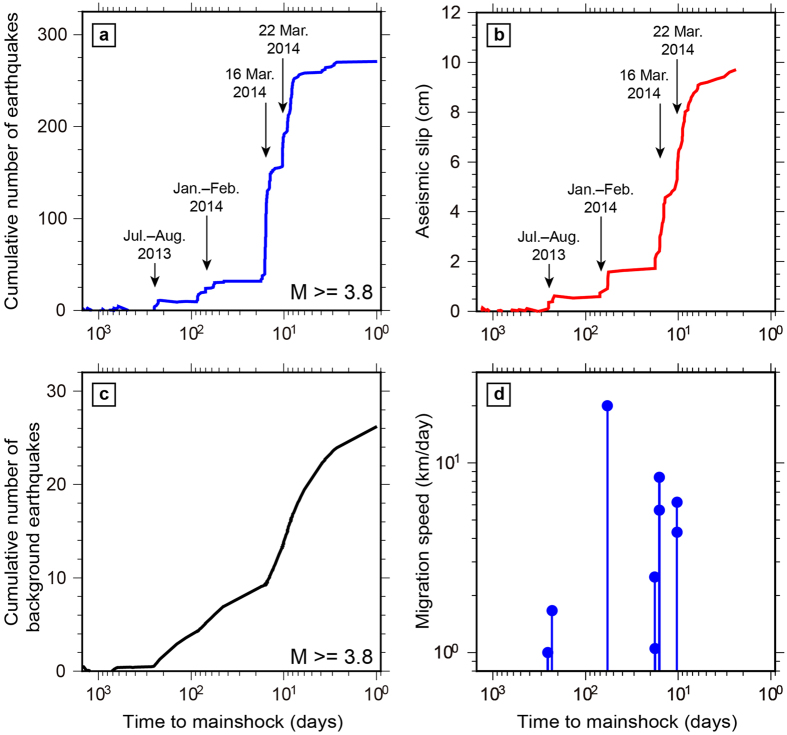
Accelerated fast and slow slip during the nucleation stage. (**a**) Detrended cumulative number of earthquakes, (**b**) detrended aseismic slip averaged over all groups of repeating earthquakes, (**c**) detrended cumulative number of background seismicity, and (**d**) earthquake migration speeds, as a function of time to failure during the final 1200 days before the 2014 Iquique, Chile Mw 8.2 earthquake.

The displacement of aseismic slip (Figs 2c and 3b) was calculated based on a unique scaling relationship between seismic moment and fault slip for repeating earthquakes along the San Andreas Fault^45^. This scaling relationship has been applied to repeating earthquakes around the Japanese islands and the eastern Taiwan to estimate slip rates along major plate boundary faults^17^,^19^. Because slip rate distributions correlate well with coupling patterns obtained from inversion of geodetic measurements, we believe that this scaling law is suitable for the present study area. Aseismic slip estimates in the whole studied region were totaled and the sum was divided by the number of groups of repeating earthquakes identified in this study (Fig. 2c).

**Figure 4:**
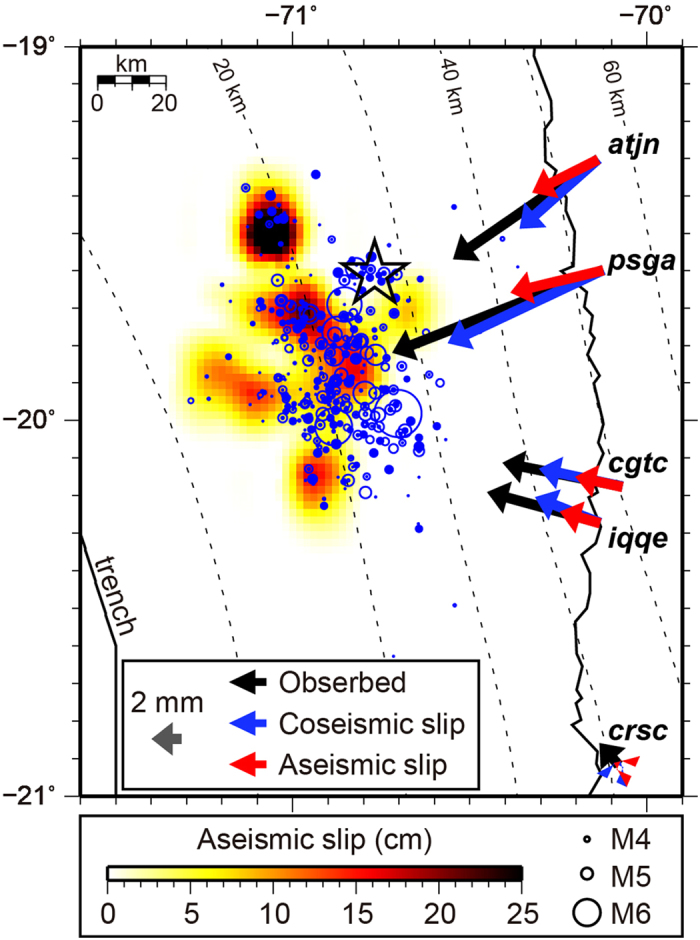
Surface deformations during the final 17 days before the 2014 Iquique, Chile Mw 8.2 earthquake (15–31 March, 2014). Black arrows denote horizontal surface displacements measured at continuous GPS stations located close to the foreshock region^10^. Blue and red arrows indicate horizontal surface displacements at each GPS station predicted from the cumulative coseismic slip of foreshocks^10^ and from aseismic slip extracted from repeating earthquakes (this study), respectively. Color scale represents the amount of aseismic slip. Open star shows the epicenter of the mainshock, and blue circles scaled to magnitude denote the epicenters of foreshocks during 15–31 March 2014. Map was created using the GMT software package^51^.

To project the aseismic slip onto the plate interface, we first estimated the aseismic slip distribution during the final 17 days before the mainshock (15–31 March, 2014) by averaging the cumulative slip of different groups of repeating earthquake located within 0.15 × 0.15° grids shifted in 0.075° increments (Fig. 4). We then projected the repeater-inferred aseismic slip on the plate interface using Slab 1.0 geometry^46^ and predicted the surface deformations caused by repeating earthquakes. The curved plate interface is modeled as a collection of triangular dislocation elements in a homogeneous elastic half-space^47^. We assumed Poisson’s ratio to be 0.25.

It could be argued that the assumed stress drop assumed for the extraction of repeating earthquakes could affect the evolution of aseismic slip. To investigate the sensitivity of the assumed stress drop on repeater-inferred aseismic slip, we analyzed two circular patch models with different assumed stress drops: 1 MPa (Supplementary Table S2) and 10 MPa (Supplementary Table S3). Based on the assumed stress drop, we adjusted the frequency bands for cross-correlation computations, and calculated the rupture radii of each event pair as a colocation check. The resultant spatial–temporal evolutions of aseismic slip (Supplementary Figs S8 and S9) show similar behaviors to the 3 MPa case (Fig. 4). Although the repeater-inferred surface deformations depend slightly on the assumed stress drop, the difference is not significant enough to change our conclusions.

### Calculation of background rate of seismicity

We applied the nonstationary ETAS model^23^,^24^ to fit the earthquake occurrences with magnitude greater than 3.8 (completeness magnitude *M*_*c*_). Among the parameters (*μ*, *K*_0_, *α*, *c*, *p*) of the ETAS model, the background rate of seismicity *μ* and the aftershock productivity *K*_0_ are most sensitive to the sources of non-stationarity^23^,^24^,^48^. Thus, the parameters for the background rate *μ* and the aftershock productivity *K*_0_ in the ETAS model


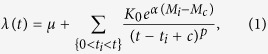


are modified to be time dependent in such a way that *μ*(*t*) and *K*_0_(*t*) should be related to the crustal stress changes caused by aseismic transient deformation and triggering effect by earthquakes, respectively. Note that any potential changes to the other parameters are absorbed by the temporal change in *K*_0_(*t*) in our model. We first estimated the three parameters *α*, *c* and *p* of the ETAS model using the earthquake data from May 2009 to June 2013, where the seismicity rate had been almost constant (stationary) (Fig. 2b): the values obtained in this model were *c* = 9.76 × 10^−5^ (±0.89 × 10^−5^), *α *= 0.987 (±0.103), *p* = 0.866 (±0.081), respectively. Fixing these three parameters (*α*, *c*, *p*) to be constant, we inverted earthquake occurrence for the optimal solution of the time variable parameters *μ*(*t*) and *K*_0_(*t*) under proper smoothness constraints with the assumption that the aseismic stress and the changes in the triggering parameter both evolve smoother than the seismicity rate changes. We then applied smoothness constraints to prevent arbitrarily rough variations in *μ*(*t*) and *K*_0_(*t*) which were not justified by the data. Defining the roughness penalty functions, Φ_*μ*_ and Φ_*K*_, as the sum of squares of the gradients in the respective parameters, the penalized log-likelihood against the roughness becomes


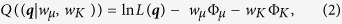


where ***q*** = (*μ*(*t*)*, K*_0_(*t*)). ln *L*(***q***) represents the log-likelihood function of the point process, and the weight parameters *w*_*μ*_ and *w*_*K*_ are the weights of the smoothness constraints. Rather than using *ad hoc* values, we selected optimal weights for the inversion via the Akaike Bayesian Information Criterion^49^. Our algorithm^23^ stably converges to the global maximum in *Q* as a result of the linearized functions of ***q***.

## Additional Information

**How to cite this article**: Kato, A. *et al.* Accelerated nucleation of the 2014 Iquique, Chile Mw 8.2 Earthquake. *Sci. Rep.* **6**, 24792; doi: 10.1038/srep24792 (2016).

## Supplementary Material

Supplementary Information
