# Supplementary material for: Accelerated nucleation of the 2014 Iquique, Chile Mw 8.2 Earthquake
Source: Sci Rep. 2016 Apr 25;6:24792. doi: 10.1038/srep24792 (PMC4842989; doi:10.1038/srep24792)
Supplement: Supplementary Information [file srep24792-s1.pdf]

Supplementary Information

Accelerated nucleation of the 2014 Iquique, Chile Mw 8.2 Earthquake

Aitaro Kato[1,2,*], Jun'ichi Fukuda[2], Takao Kumazawa[3], and Shigeki Nakagawa[2]

1: Earthquake and Volcano Research Center, Graduate School of Environmental Studies, Nagoya University, Nagoya, Japan.

2: Earthquake Research Institute, University of Tokyo, Tokyo, Japan.

3: The Institute of Statistical Mathematics, Tachikawa, Japan.

*corresponding. aitaro@seis.nagoya-u.ac.jp

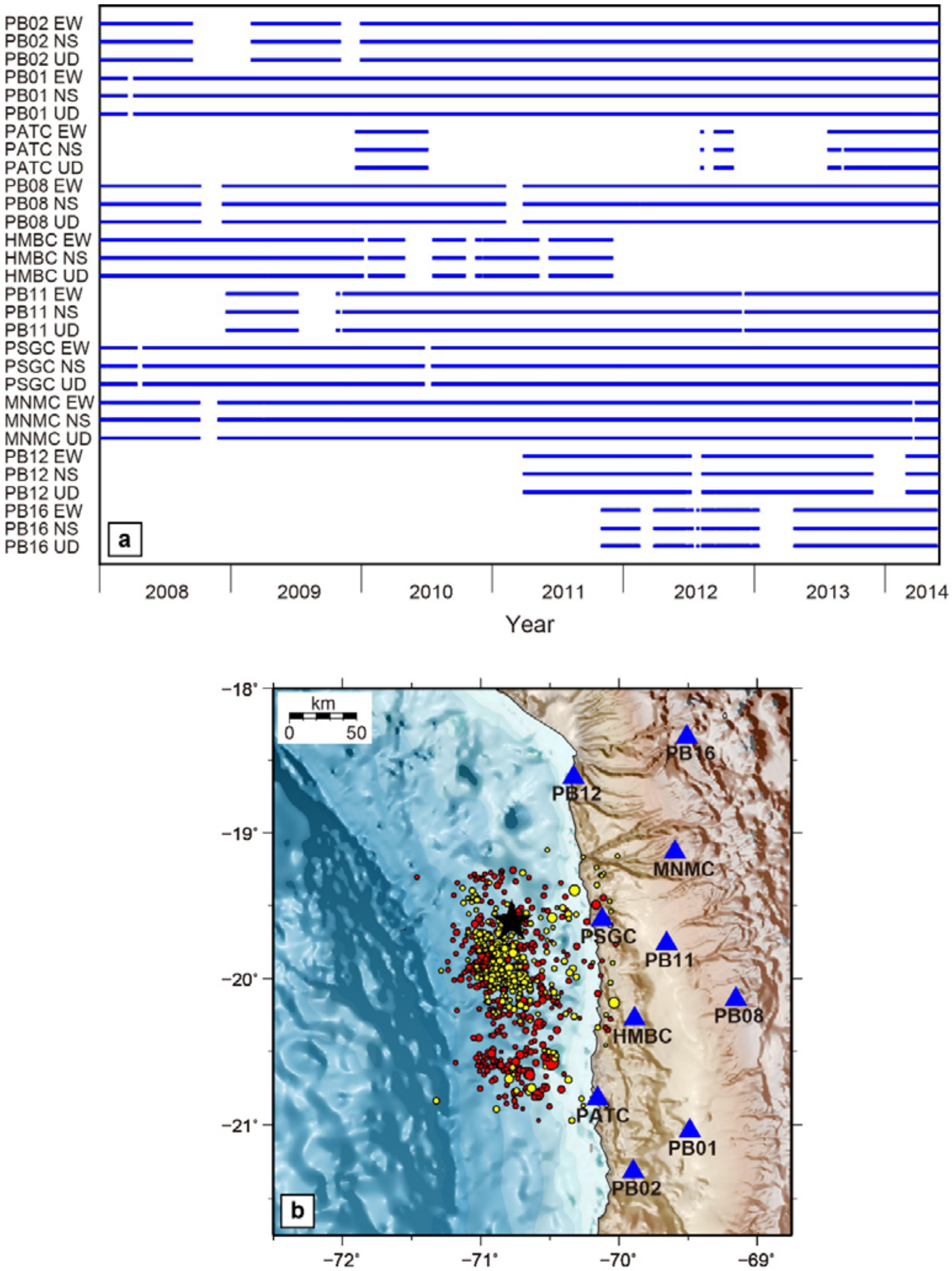

Supplementary Figure S1. Temporal availability of seismograms for each station and component. (a) Blue squares denote days when seismic waveform data were available for at least 20 hours in total. Although some stations experienced intermittent outages, most events were recorded simultaneously by more than fifteen channels (five stations). (b) Map showing the distribution of seismic stations (blue triangles). Yellow and red circles are epicenters of matched filter template events before and after the mainshock, respectively. Map was created using the GMT (Generic Mapping Tools, http://gmt.soest.hawaii.edu/) software package[1].

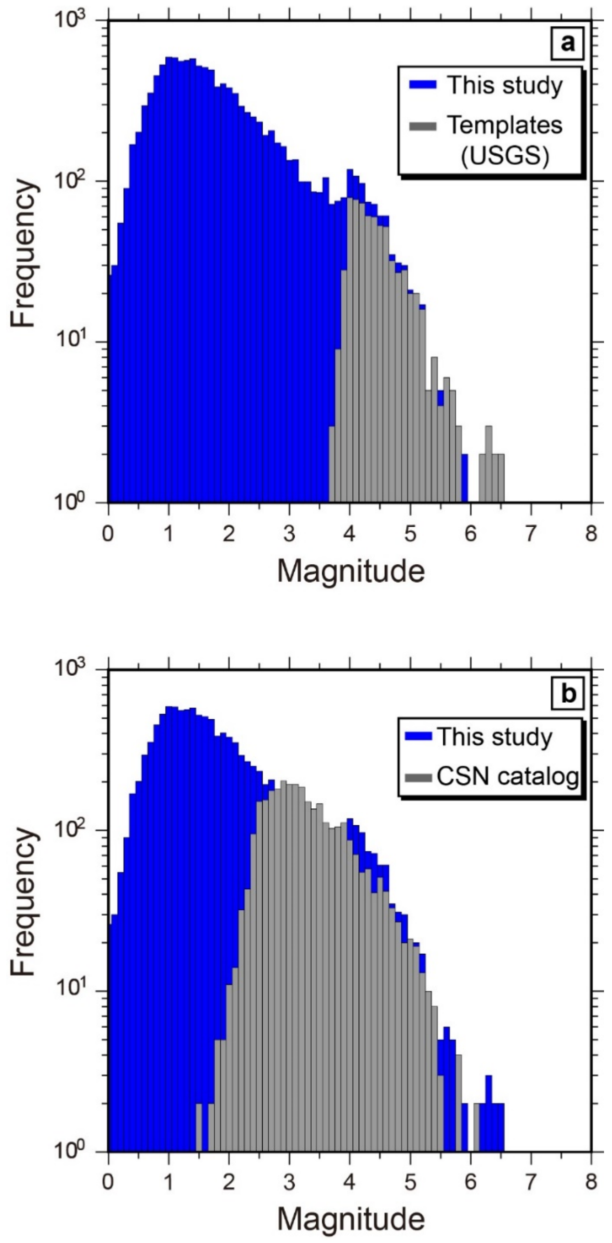

Supplementary Figure S2. Magnitude–frequency distributions of catalog earthquakes and matched filter detections. (a) Blue and gray bars represent events detected by matched filtering in the present study, and earthquakes listed in the USGS catalog (template earthquakes), respectively. (b) Blue and gray bars represent events detected by matched filtering in the present study, and earthquakes listed in the Centro Sismológico Nacional (CSN) catalog (local magnitude).

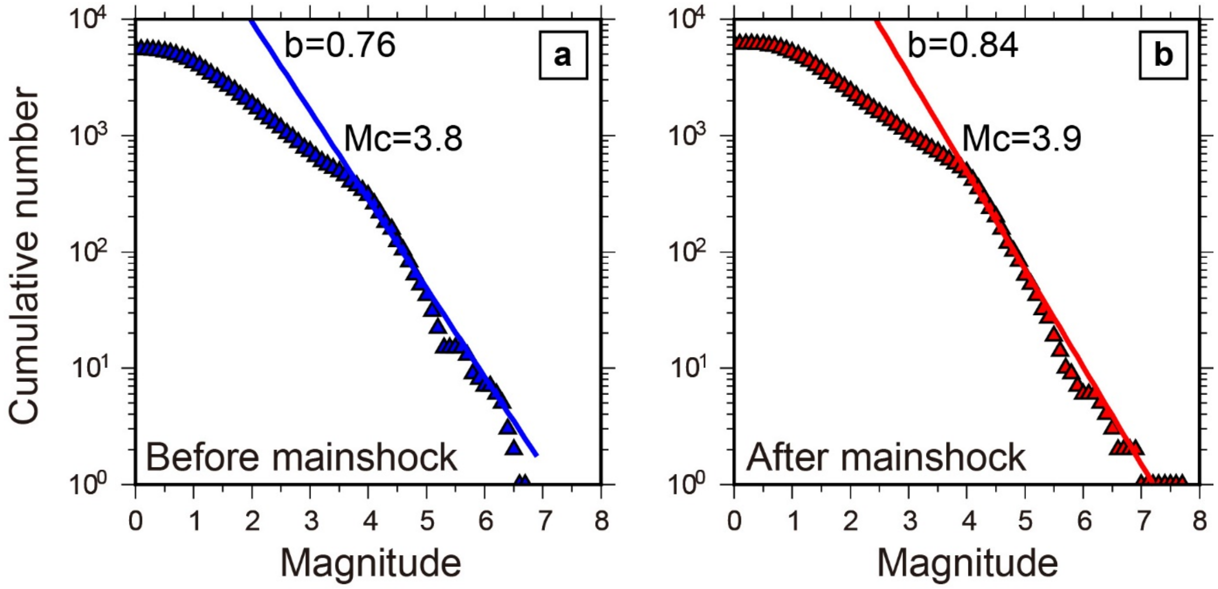

Supplementary Figure S3. Cumulative number of earthquakes as a function of magnitude for the detected events by the matched filtered technique in the present study. (a) Before the 2014 Iquique, Chile Mw 8.2 earthquake. Mc is the completeness magnitude estimated from the goodness-of-fit method[2]. The constant b-value is used to describe the relative occurrence of large and small events. (b) After the 2014 Iquique, Chile Mw 8.2 earthquake.

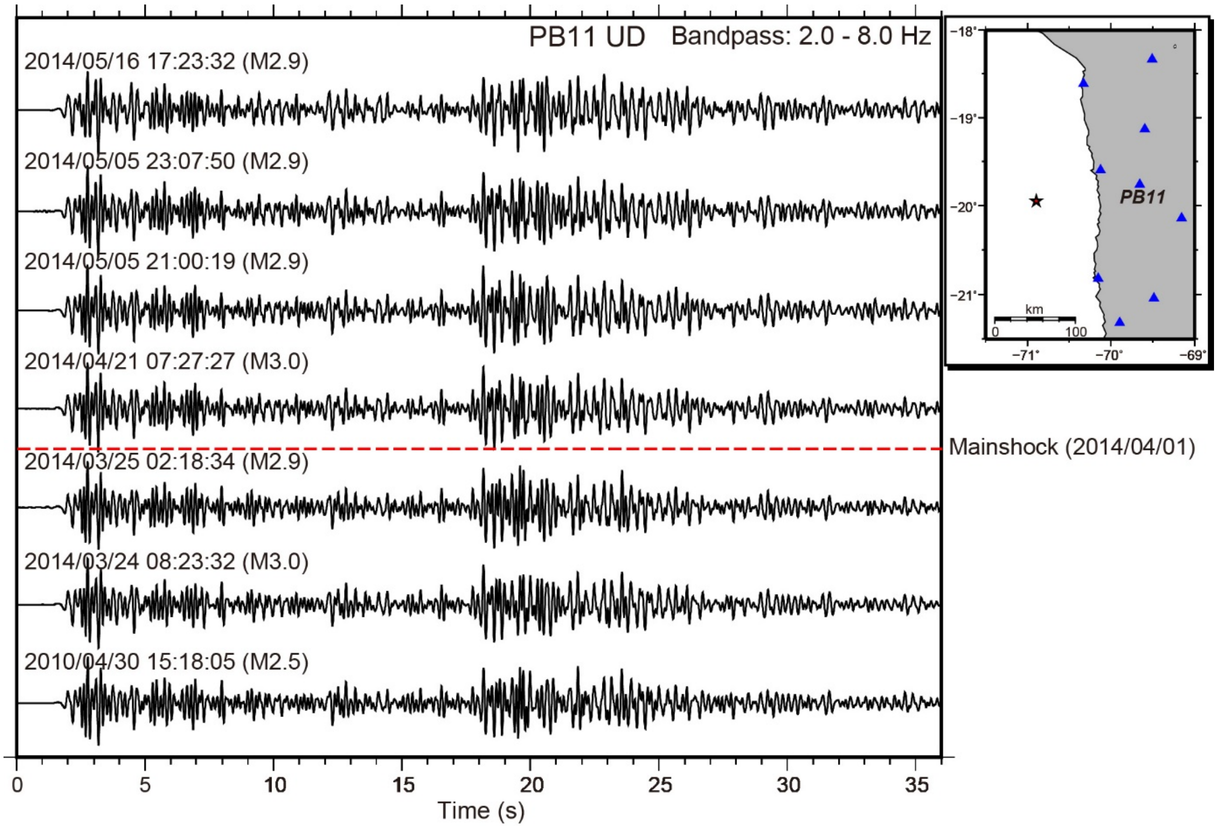

Supplementary Figure S4. Example waveforms for a group of repeating earthquakes: vertical component seismograms recorded at station PB11 are bandpass filtered from 2.0 to 8.0 Hz. The horizontal red dashed line indicates the timing of the mainshock rupture. The inset shows the epicenter of the group of repeating earthquakes (red star) and the distribution of seismic stations (blue triangles). Map was created using the GMT software package[1].

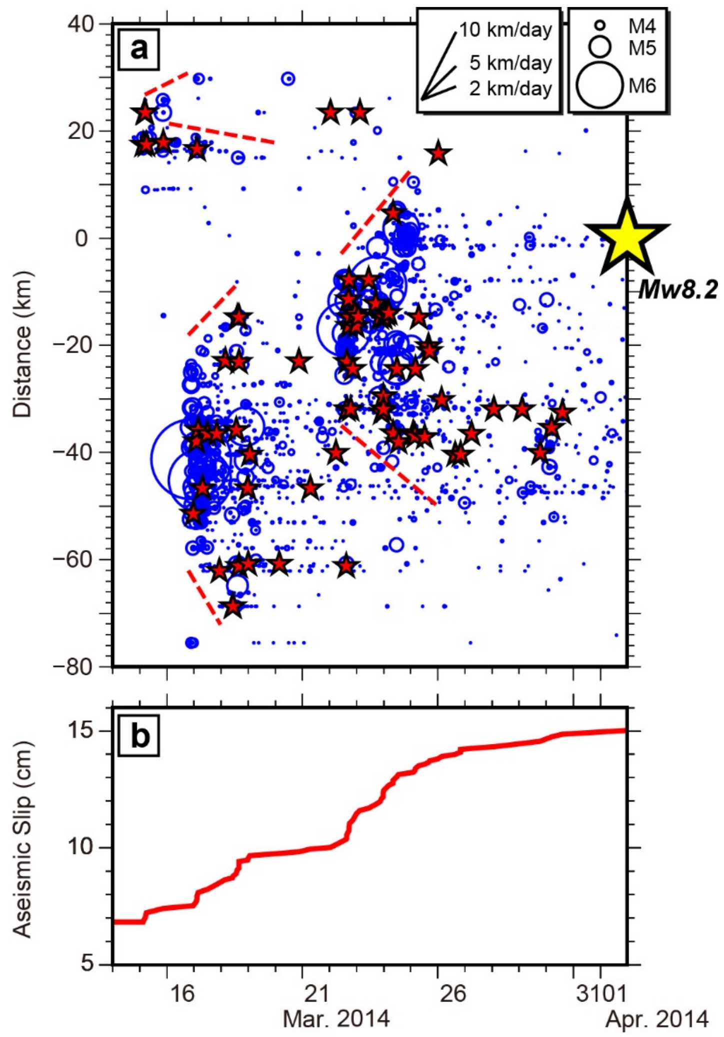

Supplementary Figure S5. Short-term evolution of seismicity during the final 17 days before the 2014 Iquique, Chile Mw 8.2 earthquake. (a) Space–time diagram of all detected events (blue circles) and repeating earthquakes (red stars). Yellow star denotes the hypocenters of the mainshock. Red dashed lines represent the approximate locations of the fronts of earthquake migrations. (b) Cumulative displacement of aseismic slip deduced from repeating earthquakes.

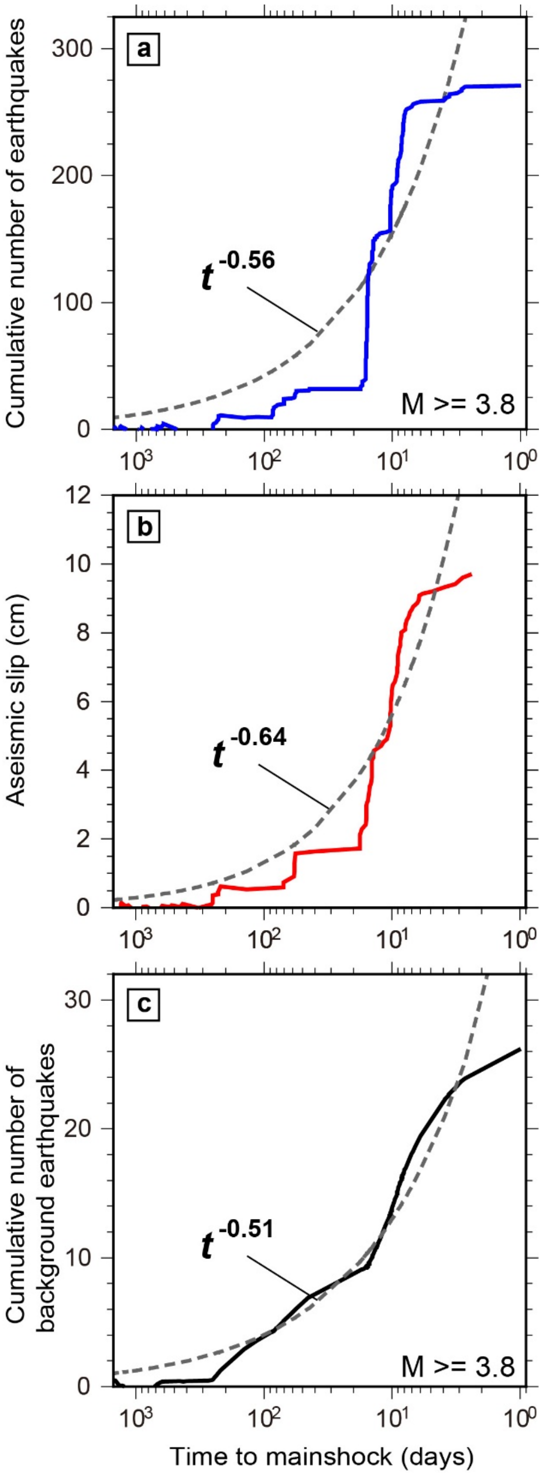

Supplementary Figure S6. Accelerated fast and slow slip during the nucleation stage, and power-law time-to-failure equations. (a) Detrended cumulative number of earthquakes. (b) Detrended aseismic slip averaged over all groups of repeating earthquakes. (c) Detrended cumulative number of background seismicity. Each dotted gray curve is the best-fit curve using a power-law time-to-failure equation.

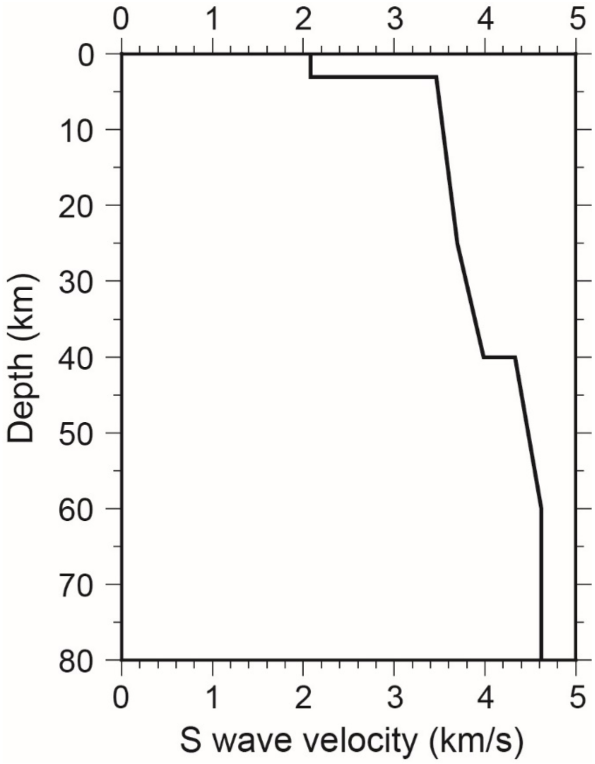

Supplementary Figure S7. One-dimensional seismic velocity structure (S wave) used in the present study.

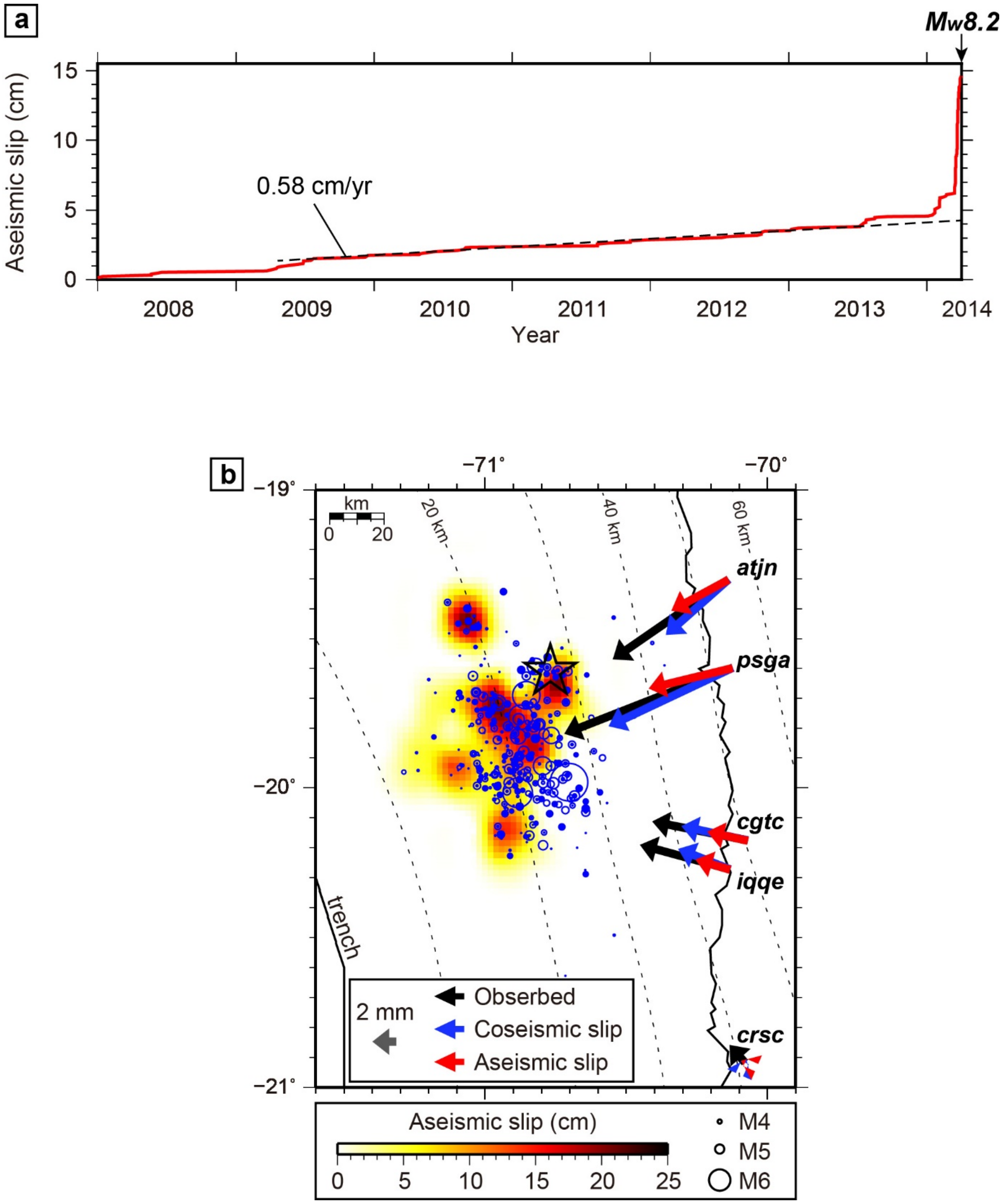

Supplementary Figure S8. Parameter sensitivity test for the extraction of repeating earthquakes (Case 1: Stress drop = 1 MPa). (a) Cumulative displacement due to aseismic slip deduced from all groups of repeating earthquakes. (b) Surface deformation during the final 17 days before the 2014 Iquique, Chile Mw 8.2 earthquake (15–31 March, 2014). For details, see the caption to Fig. 4 in the main article. Map was created using the GMT software package[1].

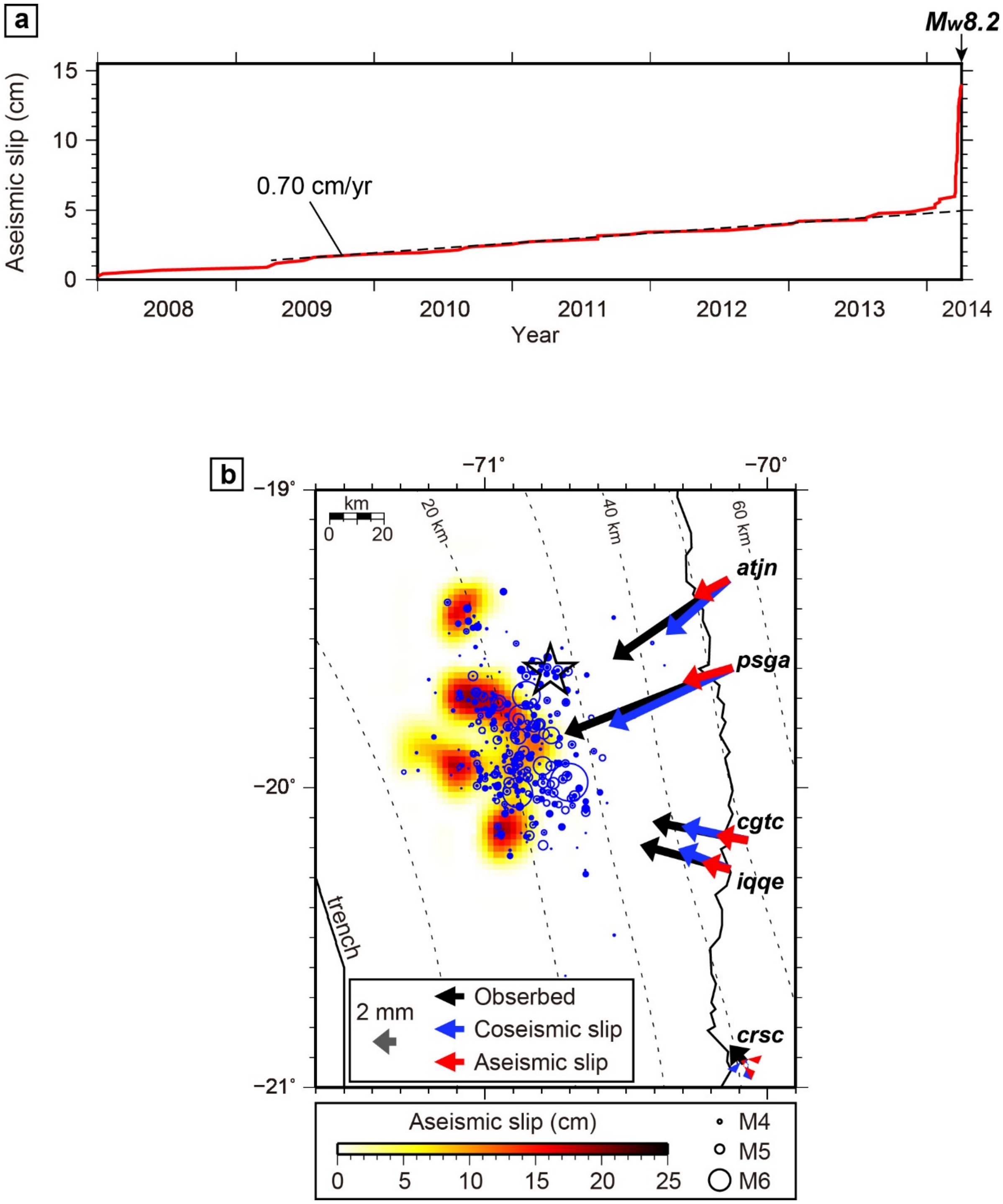

Supplementary Figure S9. Parameter sensitivity test for the extraction of repeating earthquakes (Case 2: Stress drop = 10 MPa). (a) Cumulative displacement due to aseismic slip deduced from all groups of repeating earthquakes. (b) Surface deformations during the final 17 days before the 2014 Iquique, Chile Mw 8.2 earthquake (15–31 March, 2014). For details, see the caption to Fig. 4 in the main article. Map was created using the GMT software package[1].

Range of average magnitude for an event pair	M = 2.0 – 3.0	M = 2.5 – 3.5	M = 3.0 – 4.0	M = 3.5 – 4.5
Pass-band widths of filtering	4.0 – 16.0 Hz	2.0 – 8.0 Hz	1.0 – 4.0 Hz	0.5 – 2.0 Hz
Corner frequency of an reference event (mean magnitude)	8.1 Hz (M = 2.5)	4.6 Hz (M = 3.0)	2.6 Hz (M = 3.5)	1.4 Hz (M = 4.0)
Aseismic moment released during final 17 days	1.23e+19 Nm (Mw 6.69)			

Supplementary Table S1. Relationship between the average magnitude of each event pair and filter pass-band widths in the extraction of repeating earthquakes. We calculated the reference corner frequency by assuming a circular patch model[3] with a constant stress drop of 3 MPa and S wave velocity of 3.77 km/s.

Parameter sensitivity test (case1)

Range of average magnitude for an event pair	M = 2.0 – 3.0	M = 2.5 – 3.5	M = 3.0 – 4.0	M = 3.5 – 4.5
Pass-band widths of filtering	2.0 – 14.0 Hz	1.0 – 7.0 Hz	0.5 – 3.5 Hz	0.25 – 1.75 Hz
Corner frequency of an reference event (mean magnitude)	5.6 Hz (M = 2.5)	3.2 Hz (M = 3.0)	1.8 Hz (M = 3.5)	1.0 Hz (M = 4.0)
Aseismic moment released during final 17 days	1.22e+19 Nm (Mw 6.69)			

Supplementary Table S2. Relationship between the average magnitude of each event pair and filter pass-band widths in the extraction of repeating earthquakes (Case 1). We calculated the reference corner frequency by assuming a circular patch model[3] with a constant stress drop of 1 MPa and S wave velocity of 3.77 km/s.

Parameter sensitivity test (case2)

Range of average magnitude for an event pair	M = 2.0 – 3.0	M = 2.5 – 3.5	M = 3.0 – 4.0	M = 3.5 – 4.5
Pass-band widths of filtering	8.0 – 20.0 Hz	4.0 – 10.0 Hz	2.0 – 5.0 Hz	1.0 – 2.5 Hz
Corner frequency of an reference event (mean magnitude)	12.2 Hz (M = 2.5)	6.8 Hz (M = 3.0)	3.8 Hz (M = 3.5)	2.2 Hz (M = 4.0)
Aseismic moment released during final 17 days	0.95e+19 Nm (Mw 6.62)			

Supplementary Table S3. Relationship between the average magnitude of each event pair and filter pass-band widths in the extraction of repeating earthquakes (Case 2). We calculated the reference corner frequency by assuming a circular patch model[3] with a constant stress drop of 10 MPa and S wave velocity of 3.77 km/s.

Supplementary references

1. Wessel, P. & Smith, W. H. F. New, improved version of Generic Mapping Tools released. *Eos Trans. Am. Geophys. Union* **79,** 579 (1998).
2. Wiemer, S. & Wyss, M. Minimum magnitude of completeness in earthquake catalogs: examples from Alaska, the western United States, and Japan. *Bull. Seismol. Soc. Am.* **90,** 859–869 (2000).
3. Madariaga, R. Dynamics of an expanding circular fault. *Bull. seism. Soc. Am.* **66,** 639–666 (1976).
